# COVID-19 Outbreak and Physical Activity in the Italian Population: A Cross-Sectional Analysis of the Underlying Psychosocial Mechanisms

**DOI:** 10.3389/fpsyg.2020.02100

**Published:** 2020-08-21

**Authors:** Andrea Chirico, Fabio Lucidi, Federica Galli, Francesco Giancamilli, Jacopo Vitale, Stefano Borghi, Antonio La Torre, Roberto Codella

**Affiliations:** ^1^Department of Social and Developmental Psychology, Sapienza University of Rome, Rome, Italy; ^2^IRCSS Istituto Ortopedico Galeazzi, Milan, Italy; ^3^Department of Biomedical Sciences for Health, Università degli Studi di Milano, Milan, Italy; ^4^Department of Endocrinology, Nutrition and Metabolic Diseases, IRCCS MultiMedica, Milan, Italy

**Keywords:** social distance, integrated theoretical model, confinement, pandemic, home-based exercise

## Abstract

Italy is one of the first European epicenters of the COVID-19 pandemic. In attempts to hinder the spread of the novel coronavirus disease, Italian government hardened protective measures, from quarantine to lockdown, impacting millions of lives dramatically. Amongst the enacted restrictions, all non-essential activities were prohibited as well as all outdoor activities banned. However, at the first spur of the outbreak, for about a dozen of days, physical and sports activities were permitted, while maintaining social distancing. In this timeframe, by administering measures coming from self-determination theory and theory of planned behavior and anxiety state, in an integrated approach, we investigated the prevalence of these activities by testing, via a Structural Equation Model, the influence of such psychosocial variables on the intention to preserve physical fitness during the healthcare emergency. Through an adequate fit of the hypothesized model and a multi-group analysis, we compared the most COVID-19 hit Italian region – Lombardy – to the rest of Italy, finding that anxiety was significantly higher in the Lombardy region than the rest of the country. In addition, anxiety negatively influenced the intention to do physical activity. Giving the potential deleterious effects of physical inactivity due to personal restrictions, these data may increase preparedness of public health measures and attractiveness of recommendations, including on the beneficial effects of exercise, under circumstances of social distancing to control an outbreak of a novel infectious disease.

## Introduction

Since December 2019, when a new coronavirus (SARS-CoV-2) was originally revealed by an ophthalmologist in Wuhan (Hubei province, China), a related severe acute respiratory syndrome – namely COVID-19 – has been spreading at a pandemic rate, putting global health systems under unprecedent pressure. Italy, as the first Western country tremendously hit by this disease outbreak, has become the iconic resilient outpost under international policymakers’ attention. In fact, as we write, Italy is suffering one of the deadliest impacts of coronavirus (Anderson et al., 2020). In particular, Lombardy, a region of northern Italy – the most densely populated one – has been coping with a completely different epidemiological scenario, in terms of a greater number of confirmed cases and victims, as compared to the remainder of the nation. When initial clusters were identified, restrictive actions to curb isolated upsurges of infection were taken by the health region system of Lombardy, thereafter, were extended to all northern Italy and to the entire country.

From February 21, when the first Italian COVID-19 case was diagnosed in southern Lombardy, to March 22, when Italian’s government restrictions to contain the pandemic were extended, prohibiting all non-essential business activities and banning all movements of people nationwide, the country faced an unchartered scenario, from several standpoints, along with the psychosocial ones (Bao et al., 2020). Amongst these crisis byproducts, social distancing is one of the necessary measures enabled by health authorities to nullify virus contagion due to interhuman contacts. Social distancing represents *per se* a psycho-social problem, potentially increasing mental health problems, such as depression and anxiety (Huremović, 2019), and leading to sleep and circadian disruption (Altena et al., 2020). Furthermore, quarantine and lockdown policies, not only disrupt human relationships but also foresee a revolution of one’s habits and lifestyles, including the possibility to remain physically active during a forced isolation. On a side, it is very likely that prolonged staying home (“quarantine”) might be associated with: (a) sedentary behaviors (sitting, watching tv, smart-devices activities; (b) reduced physical activity bearing low energy expenditure; and, (c) engaging in avoidance activities that, consequently, lead to an increased risk for and potential worsening of chronic health conditions (Gutin et al., 2005). On the other hand, the need to maintain regular physical activity levels is still urgent in the current COVID-19 emergency (Chen et al., 2020), even when asymptomatic SARS-CoV-2 infection has been ascertained (Joy, 2020). In fact, physical activity is capable of triggering an immune-modulatory response which is an essential forefront, on a standard basis (Codella et al., 2015), and especially under circumstances of obliged sedentariness (Codella, 2020). An enormous number of both cross-sectional and longitudinal studies have indicated that regular physical exercise exerts diversified anti-inflammatory actions (Pedersen and Saltin, 2015), offering protection against all-cause mortality (WHO, 2015). In a murine study (Lowder et al., 2005), moderate endurance exercise (30 min/day) protected mice from death due to influenza. In older adults, 10 months of moderate endurance training improved influenza vaccination responses (Woods et al., 2009) and regular exercise, in general, covers a broad spectrum of mental health benefits, from boosting mental wellness by enhancing mood states (Yeung, 1996; Berger and Motl, 2008) to reducing levels of anxiety and perceived stress (Herring et al., 2010; Codella et al., 2017). In addition, exercise and sleep have a complex and reciprocal interaction, which is explained by multiple psycho-physiological pathways, and it has been largely demonstrated that chronic moderate-intensity exercise is able to promote humans’ sleep (Chennaoui et al., 2015), On the contrary, social isolation and confinement are known to have negative effects on immunity, for instance by elevating glucocorticoids like cortisol (Cacioppo et al., 2015) and inhibiting T-cells action (Cole et al., 2015) which are vital effector lymphocytes in protecting vulnerable areas like upper respiratory tracts and lungs (Nieman, 1994).

The set of policies enacted in Italy in the time frame between March 11 (Government of Italy, 2020a), when the first official lockdown was put in place, and March 22 (Government of Italy, 2020b), when all opportunities of physical activity were abrogated, allowed people to perform a certain amount of physical activity like walking dogs, outdoor individual fitness activities (jogging, running, biking). That timespan is of particular interest as it depicted a mixture of states, motives, and modified behaviors which undoubtedly affected personal physical activity, in terms of prevalence (quantity, frequency), modality, and expectations to perform exercise and sports regardless of the pandemic period.

The present study, conducted through March 17–22, aimed at surveying Italian population on its physical activity behavior and how this latter was modeled by psychosocial variables during the emergency contingencies and measures taken for COVID-19 outbreak. As endpoint, these data might help developing targeted empirical evidence in order to strength public health policies and guidance concerning the containment of the pandemic.

In order to evaluate our aims we adopt a multi-theory, integrated approach to identify the psychological determinants of the physical activity behavior (Hagger and Chatzisarantis, 2014; Galli et al., 2018). The integrated approach encompasses multiple constructs representing key determinants and the associated processes. The integration maximizes the comprehensiveness of explanation of outcomes, assists in addressing shortcomings of single theories, and provides means to represent different processes that determine behavior (Hagger, 2009). In particular, we applied an integrated model that draws its hypotheses from two main theories of motivated action: the self-determination theory (SDT; Deci and Ryan, 1985; Ryan and Deci, 2000) and the theory of planned behavior (TPB; Ajzen, 1985). Specifically, the SDT aims to identify the contextual and environmental factors that can increase or decrease individual motivation. Central to the theory is the distinction between two main types of motivation: intrinsic and extrinsic (Ryan and Deci, 2000). Intrinsic motivation pertains to engagement in a specific activity for the pleasure and satisfaction. In contrast, extrinsic motivation refers to activities that are performed to obtain separable outcomes (Ryan et al., 2009). These motives vary along a continuum: at the lowest end there is the amotivation (when an individual does not motivate at all), and the intrinsic motivation is at the highest end (Reifsteck et al., 2016). SDT includes different types of regulations determining extrinsic motivation, each with unique characteristics: external (i.e., motivated by rewards or punishments), introjected (i.e., motivated by feeling of guilty) identified (i.e., there are important goals related to the activity) and integrated (i.e., the activity is part of who you are). SDT has been applied especially to health behaviors both in the physical activity contexts (Gutin et al., 2005; Reifsteck et al., 2016).

The TPB is a specific version of the more generalized integrated behavioral model of reasoned action approach (Fishbein, 1980). Central to this theory is the idea that the performance of one behavior is determined by behavioral intention. In turn, behavioral intention is determined by three belief-based social cognition behaviors: attitudes (favorable – unfavorable evaluations of the behavior), subjective norms (social pressure to perform the behavior) and perceived behavioral control (PBC – the beliefs people hold about resources they have to enact the behavior, and their capacity to overcome behavior related barriers). A large number of researches studied the relationships between TPB constructs and physical activity (Hagger et al., 2002; Armitage, 2005; Young et al., 2014). Results of these studies showed people are more likely to intend to engage physical activity behavior if they are positively disposed toward it (attitudes), if they perceive social pressure to do so (subjective norms), and if they believe they will be successful (PBC).

Finally, we also considered in our model state anxiety construct to evaluate how the anxious state, referred to the quarantine period, could influence the behavior inclined to physical activity through the social-cognitive predictors. In fact, state anxiety represents a cognitive process of response to stress (Spielberger, 1966). In this sense, some studies showed that state anxiety correlates negatively with activity participation (Ussher et al., 2007; Ma et al., 2008).

**FIGURE 1:**
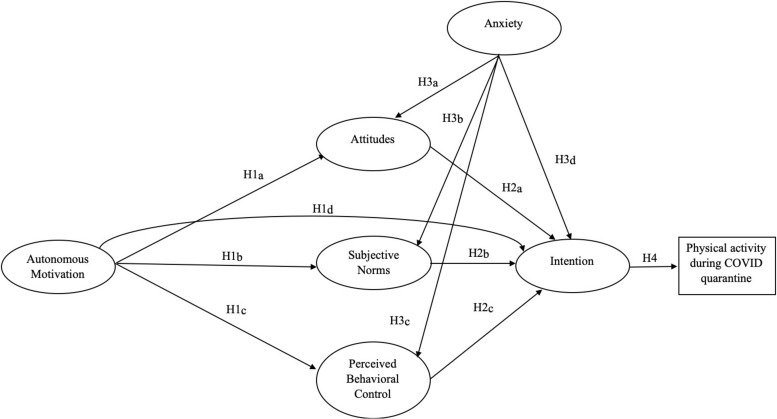
The tested hypothesis model.

With these theoretical perspectives in mind, we expected that the hypothesized integrated theoretical model would fit with the full sample of the study. In terms of specific hypotheses (Figure 1), as suggested by previous literature researches (Hagger et al., 2006, 2007; Hagger and Chatzisarantis, 2009), we expected that autonomous motivation would predict positively the TPB variables (attitudes, subjective norms, PBC, and intention – H1_a_, 1_b_, 1_c_, 1_d_); moreover, we expected that attitudes, subjective norms and PBC would positively predict the intention to do physical activity during the quarantine period (H2_a_, 2_b_, 2_c_); in turns, we expected that the higher intention would be related with a higher probability to enact the behavior (H4). Finally, we hypothesized that the state anxiety would predict negatively the behavior toward physical activity, through the mediation of the TPB constructs (H3_a_, 3_b_, 3_c_, 3_d_). Furthermore, given the specific impact that the virus had on the Lombardy region, a specific aim of this study was to evaluate specific differences between Lombardy inhabitants sample and the rest of the Italian population sample within the integrated model key variables.

## Materials and Methods

### Procedure and Participants

Data were collected via an online survey written in Italian language and administered between the 17th March 2020 to the 22nd March 2020. Participants were recruited using online advertisements. All participants were informed regards the general purpose of the study and their rights to anonymity. Researchers provided to collect written informed consent before participating in the study. The time needed to complete the survey took approximately 10 min. Collected data were coded and processed anonymously. The Department of Psychology of Development and Socialization Processes Ethical Committee of University “La Sapienza” approved the study.

### Measures

Behavior-specific version of study measures were developed specifically for the target behavior, according to the case or specifically developed from the component theories of the adopted integrated model based on previous studies (e.g., Girelli et al., 2016; Hamilton et al., 2017; Galli et al., 2018). Italian version of the measures was translated from the English version by two English-Italian bilinguals using standardized back translation procedures (Hambleton and Patsula, 1998).

#### Autonomous Motivation

The relative degree of autonomous motivation was measured using a short form of the Behavioral Regulation in Exercise Questionnaire version 3 (BREQ-3; Markland and Tobin, 2004). Participants were asked to answer on a 5-point Likert type scale (0 = “*not true for me”* and 4 = “*very true for me”*). In order to maximize the parsimony of the model in our study the relative autonomy index (RAI; Ryan and Connell, 1989) was calculated. RAI is a single score derived from the subscales that gives an index of the degree to which respondents feel self-determined. Higher, positive scores indicate greater relative autonomy; lower, negative scores indicate more controlled regulation.

#### Theory of Planned Behavior Constructs

Measures of attitudes, subjective norms, PBC, and intention from the TPB were measured using a scale developed by the authors, following the recommendations of Ajzen (1991) for TPB construct development and based on measures used in previous studies (Chirico et al., 2015; Galli et al., 2018).

In particular, each item for *attitudes* construct was introduced by “I think doing physical activity in this quarantine period is…,” comprised three items with responses provided on 7-points semantic differential scales with the bipolar adjectives “*wrong- right*,” “*disadvantageous- advantageous*,” “*useless- useful*.”

*Subjective Norms* were measured using three items by asking participants what extent meaningful others e.g., “would like me to do physical activity in this quarantine period” with responses provided on a 7-point Likert type scales (1 = “*strongly disagree”* and 7 = “*strongly agree”*). Item scores were aggregated into a single score, for which higher values indicated greater normative social pressure toward the behavior.

*PBC* was measured using three items (e.g., “I’m confident I can exercise in this quarantine period”) with responses provided on 7-point Likert-type scales (e.g., 1 = “*no control”* and 7 = “*high control”*). Item scores were aggregated into a single score, for which higher values indicated greater perceived confidence toward the behavior.

Finally, *intention* was measured using four items (e.g., “I intend to do physical activity during this quarantine period”) by asking respondents to indicate on a 7-point Likert type scale (1 = “*strongly disagree*” and 7 = “*strongly agree*”). Item scores were aggregated into a single score, for which higher values indicated greater intention toward the behavior.

*Anxiety* was measured using the six-item short form of the State-Trait Anxiety Inventory (STAI; Marteau and Bekker, 1992). Participants were asked to answer on a 6 –point Likert scale (1 = “*never*” and 7 = “*always*”; e.g., “I feel worried”).

*Self-reported behavior* was measured considering the frequency in terms of weekly hours spending on physical activity during the quarantine period. In order to evaluate the past behavior, we asked participants to report the same physical activity measure (i.e., the weekly hours spending on physical activity) during the 2 months before the quarantine period.

### Data Analysis

Statistical analyses were performed using the R language v. 3.6.3 (R Development Core Team, 2017) and the RStudio environment v. 1.2.5033 (Rstudio Team, 2019), employing a statistical significance at α = 0.05. Descriptive analyses were used to describe the sample characteristics (i.e., sociodemographic).

Relationships among the constructs were tested using structural equation modeling (SEM) through the “lavaan” package v. 0.6-5 (Rosseel, 2012). The SEM is a multivariate method that combine different analytical procedure (factor analysis and multiple regression analysis) and allow to study and assess the relationships between latent and measured variables (i.e., measurement model) and between latent variables (i.e., structural model; Gana and Broc, 2019) taking in account, at the same time, for the measurement errors. One of the assumptions to conduct SEM is the multivariate normality distribution of the data, therefore the “MVN” package v. 5.8 (Korkmaz et al., 2014) was used to assess this condition through Mardia’s multivariate normality test (Mardia, 1970). The reliability of the SEM measurement model was tested using Cronbach’s alpha (α; Cronbach, 1951) and McDonald’s hierarchical omega (ωh; McDonald, 2013). Reliability was considered “excellent” for values of Cronbach’s α ≥0.90, “good” for α between 0.90 and 0.80 and “acceptable” for α between 0.80 and 0.70 (Kline, 2013). The same thresholds values were applied for ωh (Zinbarg et al., 2005, 2006). The validity of the SEM measurement model was assessed using standardized factor loadings (i.e., measurement model coefficients), and average variance extracted (AVE). Validity was considered acceptable considering the statistical significance of the standardized factor loadings and a minimum threshold AVE value of 0.50 (Hair et al., 2010). Reliability and validity indices were calculated employing the “semTools” package v. 0.5-2 (Jorgensen et al., 2019). The indices used to assess the SEM measurement model and structural model were the Comparative Fit Index (CFI), Tucker-Lewis Index (TLI), Root Mean Square Error of Approximation (RMSEA), and Standardized Root Mean Square Residual (SRMR). Literature regards model fit indices reports a “good fit” for CFI and TLI >0.95, RMSEA <0.06, and SRMR <0.08 (Hu and Bentler, 1999). However, “acceptable fit” can be reported as long as CFI and TLI ≥0.90, RMSEA ≤0.08, and SRMR ≤1.00 (Gana and Broc, 2019). Due to the large sample size, the Chi-square (χ^2^) test and its associated significance was reported but not considered to assess the model fit (Schermelleh-Engel et al., 2003). A multi-group SEM was conducted to assess the same model in two groups, based on the region of provenance. The first group comprised people living in Lombardy, the most COVID-19 impacted region of Italy and the Italian epicenter of the disease (*n* = 1,280; “Lombardy sample”), while the second group was represented by participants from the rest of the Italian country (*n* = 1,118; “Other regions sample”). Differences across models were evaluated comparing SEM regression coefficients through z-score tests (Clogg et al., 1995; Paternoster et al., 1998). Differences between coefficients were reported as significant for *p* <0.05, employing two-tailed hypothesis. In order to perform a multi-group analysis, the SEM measurement model was previously tested for measurement and structural invariance, using multi-group confirmatory factor analysis (MG-CFA; Gana and Broc, 2019). The criteria used to assess invariance was the difference in CFI (ΔCFI) between nested models, with a threshold value of ΔCFI <0.01 (Cheung and Rensvold, 2002; Gana and Broc, 2019). Finally, we performed a series of t-test to further investigate the mean differences on key variables (i.e., intention, attitudes, subjective norms, PBC, autonomous motivation, anxiety, self-reported behavior) across the two groups. We employed Yuen’s test (Yuen, 1974) for normality and equality of variances issues, Welch’s *t*-test (Welch, 1947) only in the case of inequality of variances and Student’s *t*-tests in the event of normality and equality of variances across groups. All these tests are available in the R language and in the “WRS2” package v. 1.0.0 (Mair and Wilcox, 2019).

We assessed the power to test parameters effects (Wolf et al., 2013; Lee, 2015) employing a “proactive” Monte Carlo analyses (Marcoulides and Saunders, 2006; Marcoulides and Chin, 2013; Wolf et al., 2013) using the “simsem” package v. 0.5-15 (Pornprasertmanit et al., 2020) fixing observed variables’ standardized loadings, direct regressive paths across latent variables and correlation between attitudes, subjective norms and PBC (respectively, standardized loadings = 0.50; β = 0.40; *r* = 0.40). Moreover, we conducted an analysis to detect model misspecification in terms of RMSEA (MacCallum et al., 1996) through a *post-hoc* analysis employing the “semPower” package v. 1.0.0 (Moshagen and Erdfelder, 2016). The power level was considerate adequate if ≥0.80 (Cohen, 1992).

## Results

### Participants

Participants who responded to our survey were 2,398 in total. The demographic and descriptive characteristics and descriptive statistics of the sample and subgroups are shown in Table 1.

**TABLE 1:** Characteristics of the samples.

	Total	Other regions	Lombardy
**Age**			
*M*	31.84	34.10	29.86
*SD*	12.55	11.92	12.76
**Sex (%)**			
M	42.4	47	38.4
F	57.6	53	61.6
**Educational (%)**			
No	0.2	0.1	0.2
Primary school	0.1	0.2	0
LM school	10.4	4.2	15.8
High school	32.7	32.6	32.7
Degree or more	56.7	62.9	51.3
**House dimension (%)**			
≤50 m^2^	9.2	9.6	8.9
50–90 m^2^	42.2	41.7	42.7
≥90 m^2^	48.6	48.7	48.4
**Outdoor spaces (%)**			
Balcony	41.5	38.2	44.4
Terrace	14.2	16	12.7
Garden	35.6	35.9	35.3
No	8.7	9.9	7.7
**Numbers cohabiting (%)**			
1	14.1	16.2	11.8
2	24.8	23.8	25
3	29.1	29.3	28
4	26.7	25	27.3
5	5.3	3.9	6.3
>5	0	1.7	1.6

**M, Mean; SD, Standard Deviation; M, male; F, female; LM, lower middle.**

### Data Check Assumption

Analysis of univariate normality and descriptive statistics of items are presented in Table 2. Only attitudes items present normality issues (skewness and kurtosis > |1.96|). Regards multivariate normality distribution of the data, Mardia’s coefficient was statistically significant (*p* < 0.05). Accordingly, the SEM estimator employed was a robust version of maximum-likelihood, using Satorra–Bentler correction of chi-square and standard errors [S-Bχ^2^; (Satorra and Bentler, 2001)] and robust versions of CFI, TLI and RMSEA fit indices.

**TABLE 2:** Descriptive statistics, reliability, and validity indices of the measurement model.

Constructs	Items	Descriptive statistics				Standardized factor loadings (all *p* < 0.001)		Validity and reliability measures		
		M	SD	SK	KT	COEFF	SE	α	ω_h_	AVE
SN	SN_1_	4.04	2.05	–0.03	–1.15	0.86	0.01	0.93	0.93	0.81
	SN_2_	4.56	1.94	–0.33	–0.99	0.91	0.01			
	SN_3_	4.42	2.00	–0.25	–1.08	0.94	0.01			
PBC	PBC_1_	4.80	1.85	–0.53	–0.71	0.80	0.01	0.77	0.76	0.53
	PBC_2_	5.76	1.79	–1.41	0.89	0.55	0.02			
	PBC_3_	5.80	1.69	–1.43	1.10	0.81	0.01			
IN	IN_1_	5.79	1.86	–1.45	0.80	0.97	0.00	0.98	0.98	0.94
	IN_2_	5.67	1.93	–1.33	0.43	0.97	0.00			
	IN_3_	5.71	1.88	–1.37	0.59	0.98	0.00			
	IN_4_	5.74	1.88	–1.40	0.67	0.96	0.00			
ATT	ATT_1_	6.07	1.56	–1.98	3.05	0.90	0.01	0.89	0.89	0.73
	ATT_2_	5.95	1.59	–1.82	2.51	0.85	0.01			
	ATT_3_	6.16	1.43	–2.17	4.28	0.80	0.02			
ANX	ANX_1_	3.18	1.20	0.05	–0.70	0.87	0.01	0.81	0.81	0.45
	ANX_2_	3.04	1.10	0.31	–0.05	0.67	0.02			
	ANX_3_	2.64	1.10	0.60	0.31	0.48	0.02			
	ANX_4_	3.48	1.20	–0.08	–0.65	0.85	0.01			
	ANX_5_	3.55	1.26	–0.15	–0.60	0.66	0.01			
	ANX_6_	2.85	1.28	0.52	–0.23	0.37	0.02			
RAI	AMO	–1.25	2.99	–3.11	11.57	0.37	0.03	0.74	0.89	0.72
	EXT	–2.12	2.55	–1.41	1.52	0.20	0.02			
	INTR	4.64	3.11	0.38	–0.61	0.40	0.01			
	IDEN	20.44	4.86	–1.55	1.91	0.84	0.01			
	INTE	24.80	11.68	–0.71	–0.82	0.89	0.01			
	INTRI	34.94	12.37	–0.83	–0.04	0.87	0.01			

**M, Mean; SD, Standard Deviation; SK, Skewness; KT, Kurtosis; COEFF, coefficients; SE, Standard Error; α, Cronbach’s alpha; ωh, Hierarchical omega; AVE, Average Variance Extracted; SN, Subjective Norms; PBC, Personal Behavior Control; INT, Intention; ATT, Attitudes; ANX, Anxiety; RAI, Relative Autonomy Index; AMO, Amotivation; EXT, External; INTR, Introjected; IDEN, Identified; INTE, Integrated; INTRI, Intrinsic.**

### Power Analysis

Results regards the “proactive” Monte Carlo analysis (*N* = 1,118; 10,000 replications) exhibited an adequate average power to detect non-zero parameters (*M* = 0.98). Also, the power to detect model misspecification in terms of the RMSEA was considerate as adequate (≥0.99) with a sample of 1,118 participants.

### Measurement Model

Findings regarding the reliability and validity of the measurement model are shown in Table 2. Regarding reliability, the PBC and the autonomous motivation were acceptable (0.7 ≤ α < 0.8), the attitudes and the anxiety were “good” (0.8 ≤ α < 0.9) and the subjective norms and the intention were excellent (0.9 ≤ α). The ω_h_ values reported a difference of the reliability interpretation only for the autonomous motivation (α = 0.74; ω_h_ = 0.89), probably due to its multidimensionality and unequal factor loadings (see Zinbarg et al., 2005). Relative to validity, all items loaded on their respective latent variable in a significant way (*p* < 0.001). All constructs showed an AVE above 0.50, except for anxiety (AVE = 0.45), nevertheless, given the ω_h_ value above 0.70, the validity of anxiety was considered adequate (Fornell and Larcker, 1981). The measurement model showed good fit indices [S-Bχ^2^_(260)_ = 1734.104, *p* < 0.001; Robust CFI = 0.961; Robust TLI = 0.955; Robust RMSEA = 0.053; SRMR = 0.047].

### The Structural Equation Model

**FIGURE 2:**
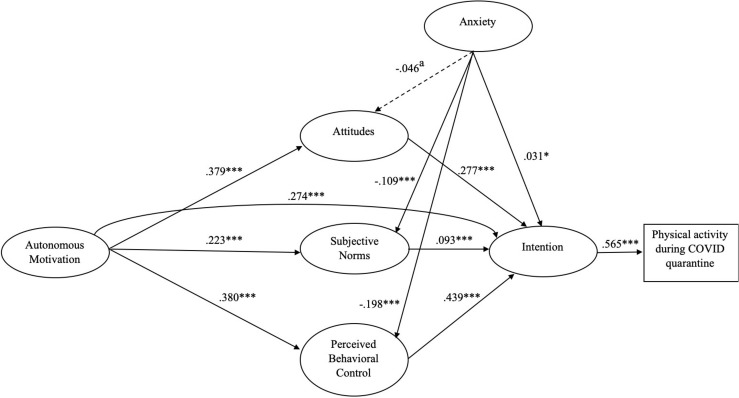
The total sample model. Results of the structural equation model for the proposed integrated theoretical model related to the total sample. Dashed lines indicate paths that were not statistically significant (*p* > 0.05). ^∗∗∗^*p* < 0.001; ^∗^*p* < 0.05; a *p* = 0.05.

The total sample model exhibited a good fit, according to the fit indices’ values [S-Bχ^2^_(284)_ = 2030.860, *p* < 0.001; Robust CFI = 0.956; Robust TLI = 0.950; Robust RMSEA = 0.055; SRMR = 0.049]. Figure 2 reports the structural model and standardized path coefficients. Regarding the relationship across autonomous motivation and TPB variables, findings report that autonomous motivation was a significant positive predictor of TPB variables; the same positive and significant effect has been founded for attitudes, subjective norms and PBC on behavioral intention. Moreover, anxiety negatively affected subjective norms and PBC, with a marginally significant effect on attitudes, meanwhile it had a positive effect on intention. Analysis of indirect effects of the total sample model (see Supplementary Appendix B – Table B1), exhibited a positive effect of the autonomous motivation on intention through attitudes, subjective norms and PBC. Conversely, anxiety significantly and negatively predicted intention through subjective norms and PBC, with also a marginally significant effect through attitudes. Lastly, to control the effect of past behavior on all the variables, a further analysis that included the physical activity behavior before quarantine period was conducted (Hagger et al., 2015). The inclusion of past behavior did not lead to a decrease of the model fit, according to the fit indices’ values [S-Bχ^2^_(304)_ = 2012.846, *p* < 0.001; Robust CFI = 0.958; Robust TLI = 0.952; Robust RMSEA = 0.052; SRMR = 0.048]. Findings showed positive and significant relationships between past physical activity behavior and autonomous motivation (β = 0.526, *p* < 0.001), intention (β = 0.038, *p* < 0.01) and physical activity during quarantine period (β = 0.439, *p* < 0.001), meanwhile a negative and significant relationship arose with anxiety (β = −0.09, *p* < 0.001). In addition, the inclusion of past behavior in the model led to a significant reduction of the intention effect on physical activity during quarantine period (*z* = −8.164, *p* < 0.001; β_past_ = 0.415 vs. β_no_past_ = 0.565) and to an increase of the variance explained by the model for the physical activity during quarantine period (from *R*^2^ = 0.319 to *R*^2^ = 0.488). For a full overview for the differences of all the effects of past behavior on all the variables, see in Supplementary Appendix B – Tables B1, B2.

### Measurement Invariance

In order to perform a multi-group analysis, a preliminary assumption is to verify the invariance of the model assessing both the measurement and the structural invariance.

The MG-CFA five steps procedure was adopted in order to assess the measurement invariance and three further stages tested the structural invariance. The first step demands a separated CFA for each subgroup to investigate the goodness of fit for each different model. Values of the fit indices measurement model for each subgroup showed satisfactory fits [Lombardy model: S-Bχ^2^_(260)_ = 918.398, *p* < 0.001; Robust CFI = 0.969; Robust TLI = 0.964; Robust RMSEA = 0.048; SRMR = 0.045; Rest of Italy: S-Bχ^2^_(260)_ = 1066.354, *p* < 0.001; Robust CFI = 0.952; Robust TLI = 0.945; Robust RMSEA = 0.058; SRMR = 0.056].

The second step requires to test a configural invariance model and to assess the fit indices. Also, this model reported a “good fit” (see Supplementary Appendix A – Table A1). From the third step onwards, various constraints were gradually added and the ΔCFI threshold was applied, to evaluate each subsequent model with the previous one. As reported in Supplementary Appendix A – Table A1, all nested models exhibited a ΔCFI <0.01, indicating that the multi-group SEM could be applied.

### Multi-Group SEM

**FIGURE 3:**
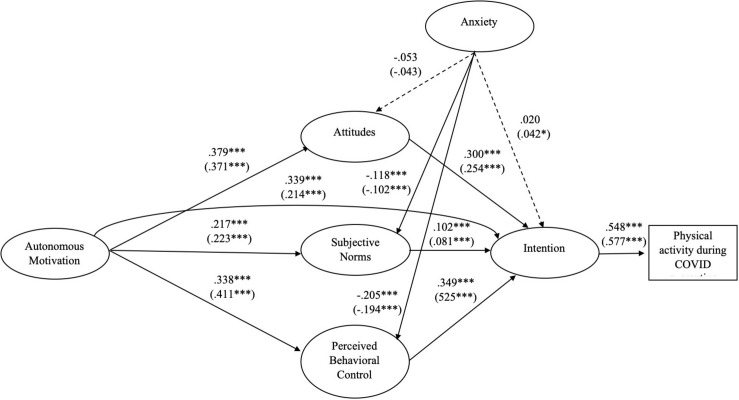
The multi-group model. Results of the structural equation model for the proposed integrated theoretical model related to the comparison between Lombardy sample and the other regions sample. Standardized path coefficients for Lombardy sample are reported in parentheses. Dashed lines indicate paths that were not statistically significant (*p* > 0.05). ^∗∗∗^*p* < 0.001; ^∗^*p* < 0.05.

The multi-group model reported a good fit [S-Bχ^2^_(608)_ = 2326.813, *p* < 0.001; Robust CFI = 0.958; Robust TLI = 0.952; Robust RMSEA = 0.052; SRMR = 0.048]. Figure 3 reports the multi-group structural model and standardized path coefficients. Regarding differences between Lombardy sample and other region sample, Lombardy group exhibited a larger effect of PBC on intention (*z* = 3.397, *p* < 0.001; β_Lom_ = 0.525 vs. β_Oth_ = 0.349), along with the indirect effect of autonomous motivation on intention through the effect of PBC (*z* = 3.389, *p* < 0.001; β_Lom_ = 0.216 vs. β_Oth_ = 0.118), while the people from other regions reported a greater effect of autonomous motivation on intention (*z* = −3.363, *p* < 0.001; β_Lom_ = 0.214 vs. β_Oth_ = 0.339). Furthermore, anxiety was a positive and significant predictor of intention only for Lombardy inhabitants (β_Lom_ = 0.042, *p* < 0.05; β_Oth_ = 0.020, *p* = 0.363). Mediation analysis of subjective norms and PBC for the relationship between anxiety and intention reported partial mediation effects for Lombardy sample and total mediation effects in the other regions group, while attitudes acted on intention via only direct effect for all subgroups (Supplementary Appendix B – Table B1).

As performed for the total sample model, the effect of past behavior on all the variables was tested also in the multi-group analysis.

The inclusion of past behavior did not lead to a decrease of the model fit for both groups according to the fit indices’ values [Lombardy sample: S-Bχ^2^_(304)_ = 1097.008, *p* < 0.001; Robust CFI = 0.965; Robust TLI = 0.960; Robust RMSEA = 0.048; SRMR = 0.045; Other regions sample: S-Bχ^2^_(304)_ = 1226.507, *p* < 0.001; Robust CFI = 0.950; Robust TLI = 0.942; Robust RMSEA = 0.057; SRMR = 0.056]. Results regarding both Lombardy sample and other regions sample exhibited a positive and significant effect of past behavior on autonomous motivation (β_Lom_ = 0.540, *p* < 0.001) and current physical activity (β_Lom_ = 0.415, *p* < 0.001), and a negatively significant effect on anxiety (β_Lom_ = −0.069, *p* < 0.05). Furthermore, only in the other regions sample the past behavior showed a positive and significant effect on intention (β_O*th*_ = 0.048, *p* < 0.05).

Moreover, in both groups, when controlling for past behavior, the effect of intention on physical activity behavior during quarantine period decreased (Lombardy sample: *z* = −5.686, *p* < 0.001; β_past_ = 0.437 vs. β_no_past_ = 0.577; Other regions sample: *z* = −5.770, *p* < 0.001; β_past_ = 0.388 vs. β_no_past_ = 0.548), leading also to an increase of the variance explained on actual behavior (Lombardy sample: from *R*^2^ = 0.333 to *R*^2^ = 0.484; Other regions sample: from *R*^2^ = 0.301 to *R*^2^ = 0.491).

Furthermore, considering the differences between the two sub-samples on the relationships between all the variables, results showed the same differences that were present in the models without controlling for past behavior. More specifically, differences regard the relationships between autonomous motivation and intention, PBC and intention and the indirect effect of autonomous motivation on intention though PBC. For a full overview for the differences of all the effects of past behavior on all the variables in both groups and between them, see the Supplementary Appendix B – Tables B1, B3–B5.

### Means Comparison of Key Variables

In order to understand differences emerged in the multi-group analysis, a comparison of the two subgroups on all the key variables of the study has been performed.

The Table 3 reports all descriptive statistics regarding key variables across subgroups. Analysis of univariate normality reported non-normality only for attitudes in both subgroups. Levene’s test for homogeneity of variance across groups indicated unequal variances for intention, attitudes and autonomous motivation. Accordingly, Yuen’s test was used for the comparison for attitudes, the Welch’s *t*-test was employed for intention and autonomous motivation comparisons across groups meanwhile Student’s *T*-Tests were used for other variables. Results showed that the anxiety score was significantly higher in people living in Lombardy region (*M* = 18.96, *SD* = 5.21) compared to people living in other regions [*M* = 18.47, *SD* = 5.09; *t*_(2396)_ = 2.33, p < 0.05]. Autonomous motivation [*t*_(2365.8)_ = 4.35, *p* < 0.001], intention [*t*_(2382.5)_ = 3.29, *p* < 0.01], attitudes [*t*_(1376.59)_ = 3.61, *p* < 0.001], subjective norms [*t*_(2396)_ = 2.21, *p* < 0.05], and physical activity behavior [*t*_(2396)_ = 2.18, *p* < 0.05] were significantly higher in the other regions sample than Lombardy one. PBC mean differences across groups was not significant [*t*_(2396)_ = 0.938, *p* = 0.35].

**TABLE 3:** Descriptive statistics of key variables across sub-samples.

	Lombardy sample				Other regions sample				
	M	SD	SK	KT	M	SD	SK	KT	t
Autonomous Motivation	79.01	29.90	–0.77	–0.29	84.27	29.23	–1.15	0.60	4.35***
Attitudes	5.96	1.46	–1.70	2.29	6.17	1.28	–2.02	3.96	3.61***
Subjective Norms	4.26	1.89	–0.19	–1.02	4.43	1.84	–0.24	–0.94	2.21*
PBC	5.43	1.44	–0.99	0.35	5.48	1.51	–1.06	0.41	0.94
Intention	5.61	1.89	–1.29	0.36	5.86	1.78	–1.57	1.21	3.29**
State anxiety	18.96	5.21	0.10	–0.23	18.47	5.09	0.13	–0.23	2.33*
PA during COVID quarantine	2.61	2.15	0.28	–1.27	2.8	2.16	0.19	–1.33	2.18*

M, Mean; SD, Standard Deviation; SK, Skewness; KT, Kurtosis; PA, Physical Activity; t, t-test. *** p < 0.001; ** p < 0.01; * p < 0.05.

## Discussion

The Italian Government implemented extraordinary measures to limit viral transmission of the COVID-19 since the 8th March 2020. These actions included, firstly, the restriction of people movement. Gradually, Italian Government decreed stricter measures in order to minimize the virus transmission until reaching 22nd March 2020, date on which a total lockdown of all the commercial and recreational activities, including sports ones, was ordered, thus, obliging people to radically change their lifestyles also in terms of physical activity. Possible consequences of widespread outbreaks of infectious diseases, such as COVID-19, and the harsh measures adopted to prevent these infections are associated with psychological distress and symptoms of mental illness (Hawryluck et al., 2004; Anderson et al., 2020; Bao et al., 2020).

The main aim of our study was to evaluate the role of different psychosocial predictors of physical activity, during the unique context of pandemic diffusion of COVID-19. In light of this, we tested an integrated theoretical model in Italian population in order to understand the psychosocial constructs underpinning the physical activity behavior.

In line with our purpose, we firstly tested an integrated behavioral model linking autonomous motivation, attitudes, subjective norms, PBC and anxiety, with the intention to do physical activity during quarantine, and in turns, the relationship between the intention and the behavior itself. The hypothesized model showed a good fit with our data.

Considering the full sample of the Italian population, a first tested hypothesis was the link between autonomous motivation and TPB variables. Our results showed that, during the lockdown for COVID-19, individuals whose motivation to enact physical activity is self-determined (autonomous motivation), have positive attitudes toward the physical activity (H1_a_; attitudes), they feel supported by their “important others” (H1_b_; subjective norms), and, since their motivation is self-determined, they feel the possibility to do physical activity under their perceived control (H1_c_; PBC). Conversely, people who are not motivated, or whose motivation is external would have, accordingly, worst attitudes, would feel less supported and lower PBC. Our tested hypotheses are in line with literature dealing with the integration of SDT and TPB suggesting that motivation to engage in health-related behaviors for self-determined or external reasons (e.g., sense of guilty, medical condition, physicians suggestions) predisposes individuals to form beliefs congruent with these motives (Hagger and Chatzisarantis, 2009), and that self-determined motivation can be supported or thwarted by environmental contingencies (Reeve et al., 1999; Hagger and Chatzisarantis, 2007).

Our results, therefore, showed that autonomous motivation has both a direct and significant effect on intention (H1_d_) and via the mediation of TPB predictors (see Supplementary Appendix B – Table B1 for indirect effects). Although the indirect effect of autonomous motivation on intention and health-related behaviors has been frequently reported by scholars, the direct effect of the autonomous motivation on intention and health-related behaviors suggests more impulsive and less deliberative processes by which self-determined motives predict intention formation and enactment (Chatzisarantis et al., 2003; Hagger et al., 2005, 2006). To speculate, the intention to enact physical activity can be considered a highly self-determined and low deliberative process, especially during the Italian lockdown policy where all sport facilities (e.g., gyms, sport fields) are closed.

Furthermore, moving forward to the second set of tested hypotheses, behavioral intention has been significantly predicted by attitudes (H2_a_), subjective norms (H2_b_), and PBC (H2_c_). In fact, different reviews and meta-analyses of literature provided robust evidence for these relationships (Sheeran and Taylor, 1999; Armitage and Conner, 2001; Sheeran et al., 2001; Hagger et al., 2002; Trafimow et al., 2002; Rivis and Sheeran, 2003; Schulze and Whittmann, 2003; McEachan et al., 2011).

Specifically, in the meta-analysis of McEachan et al. (2011), authors reported as attitudes and PBC were the strongest predictors of behavioral intention. In a similar fashion, our results indicate that PBC and attitudes influenced the intention to enact physical activity during COVID-19 pandemic with stronger effects compared to the subjective norms. Following the recommendation of other scholars (Ma et al., 2008), we implemented a measure of anxiety in order to understand its role within the hypothesized model. As expected, our data showed that anxiety had a significant negative effect on all the TPB predictors of intention, and a small unexpected positive direct effect on intention. This last effect could seem not supporting our hypothesis, since we tested the role of anxiety as inhibitor of physical activity, as suggested by other scholars dealing with this issue (Ma et al., 2008).

Currently, the literature is not consistent about the role of anxiety. In fact, different studies show that regular physical activity brings benefit to individuals with mental disease, such as depressive and anxiety symptoms (Martinsen et al., 1989; Petruzzello et al., 1991; Peluso and Guerra de Andrade, 2005).

On the other hand, part of literature focuses on the role of negative influence of the anxiety on the physical activity behavior (e.g., Ma et al., 2008; DeWolfe et al., 2020). However, following the latter theoretical perspective, we tested attitudes, subjective norms and PBC as mediators in the relationship between anxiety and intention (H4; Ma et al., 2008). Findings exhibited the role of mediator of all the tested variables, showing a significant negative effect of anxiety on the proximal predictors of the intention. To explore more deeply the unexpected positive role of anxiety on intention, we also tested a single direct effect of anxiety on intention without any mediating path. The relationship between these two variables resulted in a not significant effect (β = 0.013, *p* = 0.329), partially in line with our hypothesis. It is likely that the reason for the negligible positive effect of anxiety on intention is due to the large number of participants, as *p*-value is influenced by sample size (Kalinowski and Fidler, 2010).

A secondary aim was to apply the hypothesized model comparing participants living in the most heavily affected area in the northern of Italy (Lombardy; Percudani et al., 2020) with the rest of Italian country, within a multi-group approach. Noteworthy, it is important to underline the different number of people hospitalized for COVID-19 of the sub samples. Indeed, for each subgroup we calculated the trend of the ratio between the number of people hospitalized and the respective residence population. Considering the survey administration period (from 17th March to 22nd March), Lombardy region had the highest prevalence rate than the rest of Italy, starting with nearly 69 hospitalized per 100,000 people (other Italian regions around 12 per 100,000) and ending with approximately 94 per 100.000 (other Italian regions almost 21 per 100,000; Italian National Institute for Statistics (ISTAT), 2019; Presidency of the Council of Ministers - Italian Civil Protection Department, 2020).

Interestingly, some results are worth mentioning.

For instance, participants living in Lombardy experience a greater impact of their PBC on the intention to do physical activity, along with the indirect effect of autonomous motivation on intention through the effect of PBC, and a lower direct effect of autonomous motivation on the intention. To speculate, while in the other Italian regions a self-determined motive to do physical activity act as a direct and immediate proxy for the action, in an emergency context such as Lombardy autonomous motivation fosters a more reflexive and deliberative decision. In other words, these data suggest that people living in Lombardy region, even if highly self-motivated, could work out or train only after feeling themselves able to enact that behavior, thus, their motivation *per se* could not be enough.

Furthermore, anxiety is a small positive and significant predictor of intention only for Lombardy group, but this effect, as already stated for the model with all participants, hides an indirect pathway of the anxiety trough the TPB predictors of intention, in both groups.

For a better understanding of Lombardy region situation, differences between Lombardy inhabitants and the participants from other regions have been evaluated also trough *t*-test analysis. Results from this comparison showed a distinct situation for the individuals living in Lombardy. Firstly, people from Lombardy were living that peculiar healthcare situation, considering contagion ratios that could have a crucial impact on their mental state (Blakey et al., 2015; Percudani et al., 2020), resulted with a significant higher level of anxiety than the individuals living in other regions. Conversely, autonomous motivation to do physical activity, attitudes, subjective norms, intention, and the time spent in doing physical activity during quarantine were lower in Lombardy group than the inhabitants from other regions. These results depict a noteworthy situation in Lombardy and should be taken into account by National policies and other scholars, for specific studies focus on the mental health of people living in the hardest hit places by COVID-19, around the world.

Such peculiar epidemic contexts provide particular tool for psychosocial analysis.

In a recent review on psychological impact of quarantine, Brooks et al. (2020) reported that experiencing epidemic outbreaks can induce post-traumatic states such as stress, depression and/or confusion, among others. The authors suggested as stressor factors longer quarantine duration, infection fears, frustration, boredom, financial loss, inadequate information and supplies, stigma (Brooks et al., 2020). The sources of anxiety for quarantined and socially confined areas are obvious. On the contrary, it is not likewise expected that state anxiety would affect everyone in the same way. Here, we found that anxiety plays a major role and negatively predicts physical activity through the mediation of TPB variables, especially in Lombardy region. Undoubtedly, when it comes to exploring effects of quarantine periods on mental health and psychological well-being, practice of physical activity ought to be taken into consideration. We conducted this survey across a definite interval of the Italian outbreak of COVID-19, during which sports and outdoor physical activities were partially permitted, provided that 1-meter distance could be maintained as a safety interpersonal measure. All other sports events and competitions were postponed or canceled. Therefore, under these worryingly turbulent circumstances, the beneficial effects of exercise could have been continuously exploited. Not only maintaining recommended levels of physical activity (WHO, 2015) offers a broad immune-metabolic protection for the majority of the population, but also sedentary behaviors, associated with forced lockdown, might exacerbate the vulnerability to SARS-CoV-2. Moreover, regular exercise increases the antioxidant defense system and the immune response against microbial antigens (Zheng et al., 2015). Altogether, this body of evidence sustains the need of remaining physically active, to a legitimate extent, even at home owing to quarantine.

To our knowledge, our study is the first quantitative research showing the psychosocial mechanisms involved in the practice of physical activity, both in the Italian country and in a specific sample population extraordinarily hit by the COVID-19 pandemic, such as the northern Italian region of Lombardy.

These strengths notwithstanding, the present research has a few inherent limitations. In first place, the administration of a web-survey sets out the caveat concerning the accessibility to internet connection and the possibility to participate to the survey (Couper, 2000).

A second limit refers to the use of RAI. Indeed, the employment of this index could be controversial (Chemolli and Gagné, 2014). Although the several limits linked to the use of RAI, we used this aggregate score to guarantee a parsimonious model.

Thirdly, we evaluated two self-reported measures of physical activity behavior, a first considering a 2-month time-period (i.e., before the quarantine) and a second evaluating a short and actual time-point (i.e., during quarantine). For this reason, as future directions, a longitudinal study, might assess the hereby investigated measures during a specific time point (“post”), i.e., once the Italian government imposed stricter regulations (March 22), banning by law all people mobility nationwide.

## Conclusion

In the future, behavioral insights are warranted to guide public health policies throughout prolonged periods of isolation. In the case of highly contagious diseases, if inter-human contact must be avoided, on the other hand maintaining a physically active lifestyle while taking precautions appears a simple hygienic measure from both psychological and metabolic perspectives.

## Data Availability Statement

The datasets presented in this study can be found in online repositories. The names of the repository/repositories and accession number(s) can be found here: https://osf.io/wscmr/.

## Ethics Statement

The studies involving human participants were reviewed and approved by the Department of Psychology of Development and Socialization Processes Ethical Committee of University “La Sapienza” approved the study. The patients/participants provided their written informed consent to participate in this study.

## Author Contributions

All authors were responsible for drafting the manuscript and revising it critically for valuable intellectual content, and approved the version to be published.

## Conflict of Interest

The authors declare that the research was conducted in the absence of any commercial or financial relationships that could be construed as a potential conflict of interest.

## Supplementary Material

TABLE A1 Invariance analysis.

TABLE B1 Indirect effects and mediation analysis for total sample, Lombardy sample and other regions sample models including and excluding past behavior.

TABLE B2 Effects of past behavior on total sample model.

TABLE B3 Effects of past behavior on Lombardy sample model.

TABLE B4 Effects of past behavior on other regions sample model.

TABLE B5 Comparisons regards the effects of past behavior across sub-samples.

## Abbreviations

AVE: average variance extracted
BREQ: behavioral regulation in exercise questionnaire
CFI: comparative fit index
COVID-19: coronavirus disease
MG-CFA: multi-group confirmatory factor analysis
PBC: perceived behavioral control
RAI: relative autonomy index
RMSEA: Root mean square error of approximation
S-Bχ^2^: satorra-bentler correction of chi-square and standard errors
SDT: self-determination theory
SEM: structural equation modeling
SRMR: standardized root mean square residual
STAI: state-trait anxiety inventory
TLI: trucker-levis index
TPB: theory of planned behavior
Δ CFI: difference in CFI
χ^2^: chi-square.
