# Supplementary material for: COVID-19 Outbreak and Physical Activity in the Italian Population: A Cross-Sectional Analysis of the Underlying Psychosocial Mechanisms
Source: Front Psychol. 2020 Aug 21;11:2100. doi: 10.3389/fpsyg.2020.02100 (PMC7471606; doi:10.3389/fpsyg.2020.02100)
Supplement: TABLE A1 — Invariance analysis. [file Table_1.DOCX]

| *Appendix A*  Table A1. Summary of model fit indices for nested models to test invariance hypothesis | | | | | | | |  |
| --- | --- | --- | --- | --- | --- | --- | --- | --- |
| Models | S-Bχ² | df | R-CFI | R-TLI | R-RMSEA | SRMR | ΔCFI | |
| CF | 1988,901*** | 520 | .961 | .955 | .053 | .048 |  | |
| MI | 2029,638*** | 539 | .961 | .956 | .053 | .050 | .000 | |
| SI | 2133,540*** | 558 | .959 | .956 | .053 | .050 | -.002 | |
| ST | 2103,999*** | 583 | .957 | .955 | .053 | .051 | -.002 | |
| ELVM | 2131,232*** | 589 | .956 | .955 | .053 | .053 | -.001 | |
| ELVV | 2155,408*** | 595 | .956 | .955 | .053 | .060 | .000 | |
| ELVC | 2181,452*** | 610 | .955 | .956 | .053 | .058 | -.001 | |
| *Note.* *** (p-value chi-square < .001); CF = Configural model; MI = Metric invariant model;  SI = Scalar-invariant model; ST = Strict model; ELVM = Equality of latent variables means;  ELVV = Equality of latent variables variances; ELVC = Equality of the covariances between latent variables; S-Bχ² = Satorra-Bentler chi-square; df = degrees of freedom; R-CFI = Robust CFI;  R-TLI = Robust TLI; R-RMSEA = Robust RMSEA. | | | | | | | |  |
