# Supplementary material for: COVID-19 Outbreak and Physical Activity in the Italian Population: A Cross-Sectional Analysis of the Underlying Psychosocial Mechanisms
Source: Front Psychol. 2020 Aug 21;11:2100. doi: 10.3389/fpsyg.2020.02100 (PMC7471606; doi:10.3389/fpsyg.2020.02100)
Supplement: TABLE B1 — Indirect effects and mediation analysis for total sample, Lombardy sample and other regions sample models including and excluding past behavior. [file Table_2.DOCX]

| *Appendix B*  Table B1. Indirect effects of anxiety and autonomous motivation on intention for the total sample and multigroup models with the inclusion and exclusion of past physical activity behavior | | | | | | | | | | | | | | | | | | | | |
| --- | --- | --- | --- | --- | --- | --- | --- | --- | --- | --- | --- | --- | --- | --- | --- | --- | --- | --- | --- | --- |
| **Indirect effects** |  | Total sample | | |  | | Other regions sample | | | |  | Lombardy sample | | | | |  | Other regions sample  vs Lombardy sample | | |
|  |  | Past | No Past | z-test | |  | | Past | No Past | z-test | | |  | Past | No Past | z-test | | |  | Past |
|  |  | β | β |  |  |  | | β | β |  |  |  |  | β | β |  |  |  |  | z-test |
| β_RAI→ATT_ ^X^ β_ATT→INT_ |  | .104*** | .105*** | -.047 | |  | | .117*** | .114*** | .132 | | |  | .09*** | .094*** | -.179 | | |  | 1.073 |
| β_RAI→SN_ ^X^ β_SN→INT_ |  | .02*** | .021*** | .037 | |  | | .023** | .022*** | .076 | | |  | .016** | .018** | -.22 | | |  | .767 |
| β_RAI→PBC_ ^X^ β_PBC→INT_ |  | .167*** | .167*** | -.12 | |  | | .122*** | .118*** | -.138 | | |  | .213*** | .216*** | -.1 | | |  | -2.927** |
| β_ANX→ATT_ ^X^ β_ATT→INT_ |  | -.014* | -.013^a^ | -.165 | |  | | -.018 | -.016 | -.145 | | |  | -.012 | -.011 | -.09 | | |  | -.435 |
| β_ANX→SN_ ^X^ β_SN→INT_ |  | -.01*** | -.01*** | -.071 | |  | | -.012** | -.012** | -.067 | | |  | -.008* | -.008* | -.036 | | |  | -.705 |
| β_ANX→PBC_ ^X^ β_PBC→INT_ |  | -.09*** | -.087*** | -.163 | |  | | -.074*** | -.071*** | -.119 | | |  | -.104*** | -.102*** | -.101 | | |  | 1.311 |
| *Note* RAI = Relative Autonomy Index; ATT = Attitudes; INT = Intention; SN = Subjective Norms; PBC = Perceived Behavioral Control; ANX = Anxiety. *** *p* < .001; ** *p* < .01; * *p* < .05; ^a^ = .05 < p <.10 (marginally significant). | | | | | | | | | | | | | | | | | | | | |
