# Supplementary material for: COVID-19 Outbreak and Physical Activity in the Italian Population: A Cross-Sectional Analysis of the Underlying Psychosocial Mechanisms
Source: Front Psychol. 2020 Aug 21;11:2100. doi: 10.3389/fpsyg.2020.02100 (PMC7471606; doi:10.3389/fpsyg.2020.02100)
Supplement: TABLE B2 — Effects of past behavior on total sample model. [file Table_3.DOCX]

| *Appendix B*  Table B2. Effects of past behavior on total sample and differences between path coefficients | | | | | | |  |
| --- | --- | --- | --- | --- | --- | --- | --- |
| **Direct effects of past behavior on variables** | | |  | **β** |  |  | |
| Past Physical Activity | **→** | Autonomous Motivation |  | .526*** |  |  | |
| Past Physical Activity | **→** | Attitudes |  | .008 |  |  | |
| Past Physical Activity | **→** | Subjective Norms |  | .015 |  |  | |
| Past Physical Activity | **→** | PBC |  | .003 |  |  | |
| Past Physical Activity | **→** | Intention |  | .038** |  |  | |
| Past Physical Activity | **→** | Current Physical Activity |  | .439*** |  |  | |
| Past Physical Activity | **→** | Anxiety |  | -.09*** |  |  | |
| **Path coefficients controlling for past behavior** | | |  | **β** |  | **z-test** | |
| Autonomous Motivation | **→** | Attitudes |  | .373*** |  | -.151 | |
| Autonomous Motivation | **→** | Subjective Norms |  | .215*** |  | -.229 | |
| Autonomous Motivation | **→** | PBC |  | .382*** |  | .062 | |
| Autonomous Motivation | **→** | Intention |  | .252*** |  | -.841 | |
| Attitudes | **→** | Intention |  | .279*** |  | .050 | |
| Subjective Norms | **→** | Intention |  | .093*** |  | .004 | |
| PBC | **→** | Intention |  | .438*** |  | -.014 | |
| Intention | **→** | Current Physical Activity |  | .415*** |  | -8.164*** | |
| Anxiety | **→** | Attitudes |  | -.052* |  | -.161 | |
| Anxiety | **→** | Subjective Norms |  | -.112*** |  | -.095 | |
| Anxiety | **→** | PBC |  | -.205*** |  | -.194 | |
| Anxiety | **→** | Intention |  | .028^a^ |  | -.148 | |
| *Note.* PBC = Perceived Behavioral Control; *** *p* < .001; ** *p* < .01; * *p* < .05; ^a^ = .05. | | | | | | |  |
