# Supplementary material for: COVID-19 Outbreak and Physical Activity in the Italian Population: A Cross-Sectional Analysis of the Underlying Psychosocial Mechanisms
Source: Front Psychol. 2020 Aug 21;11:2100. doi: 10.3389/fpsyg.2020.02100 (PMC7471606; doi:10.3389/fpsyg.2020.02100)
Supplement: TABLE B3 — Effects of past behavior on Lombardy sample model. [file Table_4.DOCX]

| *Appendix B*  Table B3. Effects of past behavior on Lombardy sample model and differences between path coefficients | | | | | | |
| --- | --- | --- | --- | --- | --- | --- |
| **Direct effects** | | |  | **β** |  |  |
| Past Physical Activity | **→** | Autonomous Motivation |  | .540*** |  |  |
| Past Physical Activity | **→** | Attitudes |  | .029 |  |  |
| Past Physical Activity | **→** | Subjective Norms |  | .037 |  |  |
| Past Physical Activity | **→** | PBC |  | .017 |  |  |
| Past Physical Activity | **→** | Intention |  | .028 |  |  |
| Past Physical Activity | **→** | Current Physical Activity |  | .415*** |  |  |
| Past Physical Activity | **→** | Anxiety |  | -.069* |  |  |
| **Path coefficients controlling for past behavior** | | |  |  |  | **z-test** |
| Autonomous Motivation | **→** | Attitudes |  | .354*** |  | -.346 |
| Autonomous Motivation | **→** | Subjective Norms |  | .203*** |  | -.434 |
| Autonomous Motivation | **→** | PBC |  | .405*** |  | -.120 |
| Autonomous Motivation | **→** | Intention |  | .197*** |  | -.500 |
| Attitudes | **→** | Intention |  | .255*** |  | .021 |
| Subjective Norms | **→** | Intention |  | .080** |  | -.005 |
| PBC | **→** | Intention |  | .525*** |  | -.013 |
| Intention | **→** | Current Physical Activity |  | .437*** |  | -5.686*** |
| Anxiety | **→** | Attitudes |  | -.047 |  | -.090 |
| Anxiety | **→** | Subjective Norms |  | -.104*** |  | -.057 |
| Anxiety | **→** | PBC |  | -.199*** |  | -.117 |
| Anxiety | **→** | Intention |  | .040* |  | -.079 |
| *Note.* PBC = Perceived Behavioral Control; *** *p* < .001; ** *p* < .01; * *p* < .05. | | | | | | |
