# Supplementary material for: COVID-19 Outbreak and Physical Activity in the Italian Population: A Cross-Sectional Analysis of the Underlying Psychosocial Mechanisms
Source: Front Psychol. 2020 Aug 21;11:2100. doi: 10.3389/fpsyg.2020.02100 (PMC7471606; doi:10.3389/fpsyg.2020.02100)
Supplement: TABLE B4 — Effects of past behavior on other regions sample model. [file Table_5.DOCX]

| *Appendix B*  Table B4. Effects of past behavior on other regions sample model and differences between path coefficients | | | | | | |
| --- | --- | --- | --- | --- | --- | --- |
| **Direct effects** | | |  | **β** |  |  |
| Past Physical Activity | **→** | Autonomous Motivation |  | .507*** |  |  |
| Past Physical Activity | **→** | Attitudes |  | -.017 |  |  |
| Past Physical Activity | **→** | Subjective Norms |  | -.009 |  |  |
| Past Physical Activity | **→** | PBC |  | -.011 |  |  |
| Past Physical Activity | **→** | Intention |  | .048* |  |  |
| Past Physical Activity | **→** | Current Physical Activity |  | .466*** |  |  |
| Past Physical Activity | **→** | Anxiety |  | -.110** |  |  |
| **Path coefficients controlling for past behavior** | | |  | **β** |  | **z-test** |
| Autonomous Motivation | **→** | Attitudes |  | .387*** |  | .141 |
| Autonomous Motivation | **→** | Subjective Norms |  | .223*** |  | .111 |
| Autonomous Motivation | **→** | PBC |  | .348*** |  | .195 |
| Autonomous Motivation | **→** | Intention |  | .312*** |  | -.638 |
| Attitudes | **→** | Intention |  | .303*** |  | .055 |
| Subjective Norms | **→** | Intention |  | .103*** |  | .015 |
| PBC | **→** | Intention |  | .349*** |  | -.007 |
| Intention | **→** | Current Physical Activity |  | .388*** |  | -5.770*** |
| Anxiety | **→** | Attitudes |  | -.060^a^ |  | -.139 |
| Anxiety | **→** | Subjective Norms |  | -.121*** |  | -.077 |
| Anxiety | **→** | PBC |  | -.212*** |  | -.151 |
| Anxiety | **→** | Intention |  | .016 |  | -.121 |
| *Note.* PBC = Perceived Behavioral Control; *** *p* < .001; ** *p* < .01; * *p* < .05; ^a^ = .05 < *p* <.10 (marginally significant). | | | | | | |
