# Supplementary material for: COVID-19 Outbreak and Physical Activity in the Italian Population: A Cross-Sectional Analysis of the Underlying Psychosocial Mechanisms
Source: Front Psychol. 2020 Aug 21;11:2100. doi: 10.3389/fpsyg.2020.02100 (PMC7471606; doi:10.3389/fpsyg.2020.02100)
Supplement: TABLE B5 — Comparisons regards the effects of past behavior across sub-samples. [file Table_6.DOCX]

| *Appendix B*  Table B5. Path coefficients differences controlling for past behavior between other regions sample and Lombardy sample | | | | | | | | |
| --- | --- | --- | --- | --- | --- | --- | --- | --- |
| **Direct effects** | | |  | Other regions sample |  | Lombardy sample |  | **z-test** |
|  |  |  |  | **β** |  | **β** |  |  |
| Past Physical Activity | **→** | Autonomous Motivation |  | .507*** |  | .540*** |  | -1.278 |
| Past Physical Activity | **→** | Attitudes |  | -.017 |  | .029 |  | -1.035 |
| Past Physical Activity | **→** | Subjective Norms |  | -.009 |  | .037 |  | -.966 |
| Past Physical Activity | **→** | PBC |  | -.011 |  | .017 |  | -.583 |
| Past Physical Activity | **→** | Intention |  | .048* |  | .028 |  | .737 |
| Past Physical Activity | **→** | Current Physical Activity |  | .466*** |  | .415*** |  | 1.651 |
| Past Physical Activity | **→** | Anxiety |  | -.110** |  | -.069* |  | -.933 |
| Autonomous Motivation | **→** | Attitudes |  | .387*** |  | .354*** |  | .589 |
| Autonomous Motivation | **→** | Subjective Norms |  | .223*** |  | .203*** |  | .387 |
| Autonomous Motivation | **→** | PBC |  | .348*** |  | .405*** |  | -1.034 |
| Autonomous Motivation | **→** | Intention |  | .312*** |  | .197*** |  | 2.835** |
| Attitudes | **→** | Intention |  | .303*** |  | .255*** |  | .906 |
| Subjective Norms | **→** | Intention |  | .103*** |  | .080** |  | .661 |
| PBC | **→** | Intention |  | .349*** |  | .525*** |  | -3.406*** |
| Intention | **→** | Current Physical Activity |  | .388*** |  | .437*** |  | -1.719 |
| Anxiety | **→** | Attitudes |  | -.060^a^*** |  | -.047 |  | -.264 |
| Anxiety | **→** | Subjective Norms |  | -.121*** |  | -.104*** |  | -.380 |
| Anxiety | **→** | PBC |  | -.212*** |  | -.199*** |  | -.278 |
| Anxiety | **→** | Intention |  | .016 |  | .040* |  | -.839 |
| *Note.* PBC = Perceived Behavioral Control; *** *p* < .001; ** *p* < .01; * *p* < .05; a = .05 < *p* <.10 (marginally significant). | | | | | | | | |
